# Engineered Hypopharynx from Coculture of Epithelial Cells and Fibroblasts Using Poly(ester urethane) as Substratum

**DOI:** 10.1155/2013/138504

**Published:** 2013-12-22

**Authors:** Zhisen Shen, Jingjing Chen, Cheng Kang, Changfeng Gong, Yabin Zhu

**Affiliations:** ^1^Lihuili Hospital, Ningbo University, Ningbo 315211, China; ^2^The Medical School, Ningbo University, Ningbo 315211, China

## Abstract

Porous polymeric scaffolds have been much investigated and applied in the field of tissue engineering research. Poly(ester urethane) (PEU) scaffolds, comprising pores of 1–20 **μ**m in diameter on one surface and ≥200 **μ**m on the opposite surface and in bulk, were fabricated using phase separation method for hypopharyngeal tissue engineering. The scaffolds were grafted with silk fibroin (SF) generated from natural silkworm cocoon to enhance the scaffold's hydrophilicity and further improve cytocompatibility to both primary epithelial cells (ECs) and fibroblasts of human hypopharynx tissue. Coculture of ECs and fibroblasts was conducted on the SF-grafted PEU scaffold (PEU-SF) to evaluate its *in vitro* cytocompatibility. After co-culture for 14 days, ECs were lined on the scaffold surface while fibroblasts were distributed in scaffold bulk. The results of *in vivo* investigation showed that PEU porous scaffold possessed good biocompatibility after it was grafted by silk fibroin. SF grafting improved the cell/tissue infiltration into scaffold bulk. Thus, PEU-SF porous scaffold is expected to be a good candidate to support the hypopharynx regeneration.

## 1. Introduction

Hypopharyngeal carcinoma is one of common malignant tumors, accounting for 10–14% of all head and neck cancers [1]. Surgical intervention like laryngectomy is the main clinical treatment. However, laryngectomy, especially for those with severe and large area defects, often causes severe voice handicap, physical deformities, and emotional pain to the patients [2, 3]. Some biological substitutes like flaps obtained from radial forearm, anterolateral thigh, jejunum, colon, and stomach tissues are usually necessary in order to promote the surgical repair [4–8]. Unfortunately, those substitutes cannot restore the function of hypopharynx and reconstruct the complex structures surrounding the larynx and hypopharynx owing to the poor self-repair capability of this tissue [9].

Recent advances in tissue engineering and regenerative medicine have established a foundation on which the replacement of whole functional tissues or organs such as skeletal muscle, trachea, and esophagus, has been proved to be possible [10]. Zhu et al. investigated various biomaterials to be scaffold substrates for tissue engineered esophagus, for example, polyurethane, polylactide, polycaprolactone and their copolymers, and so forth [11–14]. However, nonuniform distribution of cells inside the whole 3D scaffold remains a major limitation.

Poly(ester urethane) (PEU) is a widely applied material in tissue engineering because of its good mechanical properties, biocompatibility, and biodegradability [15, 16]. It has been used to support the growth of human hypopharyngeal fibroblast and skeletal muscle cell, respectively. The good results convinced us that PEU is a good material candidate for hypopharynx tissue engineering [17, 18]. In this work, asymmetrical PEU scaffolds with surface pores ranging from 1 to 20 *μ*m in diameter on one side (micropore) and ≥200 *μ*m on the opposite side and within the bulk (macropore) were fabricated. After silk fibroin (SF), extracted from natural silkworm cocoons, was grafted onto the scaffold surface, primary epithelial cell (EC) and fibroblast digested from porcine hypopharynx tissue were seeded on surface and in bulk of SF-grafted PEU scaffold (PEU-SF). This asymmetrical, three-dimensional (3-D) scaffold was designed to mimic the extracellular matrix architecture of hypopharynx, enabling efficient EC and fibroblast distribution and infiltration. Cogrowth of these two cell types was evaluated with ungrafted PEU scaffold as the control. *In vivo* biocompatibility of the scaffold was assessed via subcutaneous implantation in Wistar rats. These studies were carried out aiming to advance scientific understanding of hypopharyngeal cell function and tissue regeneration.

## 2. Materials and Methods

### 2.1. Materials

Poly(ester urethane) (PEU, 58213, NAT022) was purchased from Estane Co., China. Paraformaldehyde and glutaraldehyde (GA) were supplied by Aladdin Reagent Co. (Shanghai, China) and were used as received. All other chemical reagents, including 1,4-dioxane, dimethyl sulfoxide (DMSO), ethanol, 1,6-diaminohexane, and n-propanol, were analytically pure and purchased from Sinopharm Chemical Reagent Co., without further purification. 4,6-Diamidino-2-phenylindole (DAPI) was purchased from Sigma-Aldrich Co., USA.

Trypsin (1 : 250, GNM) was purchased from Beijing Genosys Tech-Trading Co., Ltd. (China). Mouse anticytokeratin 14 (CK14) was purchased from Santa Cruz Biotechnology Inc. (USA). Mouse antivimentin and FITC-conjugated goat anti-mouse IgG were purchased from Wuhan Boster Bio-Engineering Co., Ltd. (China). All cell culture reagents were obtained from HyClone Laboratories Inc., (USA) unless otherwise specified.

SF was extracted from natural silkworm cocoons using a traditional method [19]. The cocoon fibers were boiled for 1 h in aqueous sodium carbonate solution (Na_2_CO_3_) (0.5 wt%) and rinsed with water to remove the sericin. The fibers were subsequently dissolved in calcium nitrate tetrahydrate (Ca(NO_3_)_2_ · 4H_2_O) at 80°C to yield a homogeneous solution (~5%, w/w) followed by water dialysis using a cellulose tubular membrane (molecular weight cutoff: 12,000–14,000, Sigma-Aldrich Co., USA) at room temperature (RT) for 3 days. Water was renewed every 4 hours in order to completely remove the salts. The resulted solution was freeze-dried to obtain silk fibroin sponge.

### 2.2. Fabrication of Porous PEU Scaffolds

PEU was dissolved in DMSO at RT to obtain a concentration of 10% (w/w) in a glass container with smooth surface. This solution with the container was placed in a freezer at −70°C for 4 h to induce DMSO crystallization. The frozen PEU was then immersed in an absolute alcohol at −20°C for 7 days to extract the solvent (DMSO) away completely. It was subsequently dried in a freeze dryer (Labconco, USA) overnight to obtain porous PEU scaffold with asymmetrical internal structure.

### 2.3. Scaffold Characterization

Scaffold morphology was observed under scanning electron microscopy (SEM). Scaffolds were quenched in liquid nitrogen to get cross-section specimens. Specimens were then sputter-coated with gold using an Auto Fine Coater (E-1010, Hitach, Japan) and observed under SEM (TM-3000, Hitachi, Japan) at an accelerating voltage of 10 kV. Pore diameters were estimated by measuring the distance of pore diameter in SEM images, with at least ten locations of each image and 3 images of each sample.

Scaffold porosity was determined using a liquid displacing method [20]. Water and ethanol were the displacing liquids. Water was used to measure the total scaffold volume (*V*0) including pore and PEU bulk due to its alienation to PEU material. Ethanol penetrates easily into the pores and does not induce scaffold's shrinkage or swelling because it is a nonsolvent of PEU. The scaffold was kept in ethanol with known volume (*V*1) in a density-measuring bottle for 5 min to allow the ethanol filling the pores. The ethanol-impregnated scaffold was removed from the bottle and the residual ethanol volume was recorded as *V*2. The porosity of the scaffold was expressed as *p* = (*V*1 − *V*2)/*V*0 × 100%.

Mechanical testing was performed with a 21.4 mm gauge length and 0.5~0.8 × 3 mm cross-section using a tensile tester (Instron 3366, USA) at the deformation rate of 20 mm/min. Four samples were tested for each scaffold type.

### 2.4. Modification of PEU Porous Scaffold

PEU scaffolds were immersed in alcohol/water (19/1, v/v) solution for 2~3 h to remove any adsorbed dirt, washed with a large amount of water, and dried in a vacuum oven for 24 h. Scaffolds were subsequently immersed in 1,6-hexanediamine/propanol solution at a concentration of 0.06 g/mL for 3 min at 37°C, washed with alcohol/water (19/1, v/v) three times, rinsed with water for 24 h at RT to remove unreacted 1,6-hexanediamine. An aminolyzed PEU porous scaffold was thus obtained. This aminolyzed scaffold was treated in 1 wt% GA aqueous solution for 3 h at RT, and rinsed with a large amount of water to remove all unreacted GA. The scaffold was subsequently incubated in 15 mg/mL SF/PBS solution for 24 h at 4°C, rinsed with water for 3 times. SF-grafted PEU porous scaffold (PEU-SF) was yielded.

### 2.5. Hydrophilicity Testing

The hydrophilicity before and after SF grafting was examined and compared using dynamic contact angle (CA) measurements on a Contact Angle Instrument (Dataphysics OCA20, Germany). Before measurements, samples were dried at 30°C under vacuum to a constant weight.

### 2.6. Primary Culture of ECs and Fibroblasts

ECs and fibroblasts were obtained from porcine hypopharyngeal tissue using the methods of enzymatic digestion and tissue explanting, respectively [21]. Both PEU and PEU-SF were sterilized in 75% ethanol aqueous solution for 2 h, rinsed in PBS for 2 d with changing PBS each 6 h. Fibroblasts (4th passage) were seeded at a density of 5.0 × 10^5^/mL in the scaffold bulk through the macropores. After 7 d, ECs (4th passage) were seeded onto the scaffold surface with micropores at a density of 5 × 10^5^/mL. Cells were cocultured for additional two weeks in Dulbecco's modified Eagle's medium (DMEM, Gibco, USA) supplemented with 10% fetal bovine serum (FBS). All cultures were kept in an incubator at 37°C and 5% CO_2_.

For cell morphological observation, cell pregnant scaffolds were fixed in 2.5% GA for 60 min, rinsed in PBS,and dehydrated through a series of graded ethanol from 50 to 100% with a step of 10%. Scaffolds were then snap-frozen and fractured in liquid nitrogen in order to observe the cell morphologies on scaffolds' surface and bulk.

### 2.7. Immunofluorescence Staining

Cells in/on PEU and PEU-SF were fixed in 4% paraformaldehyde for 10 min at RT and washed in PBS for 3 times with 5 min for each time. Samples were immersed in 0.2% Triton X-100 for 15 min at 37°C and washed in PBS for 3 times with 5 min for each time. Samples were then blocked in 10% goat serum for 20 min at 37°C followed by overnight incubation in primary mouse anti-CK14 or mouse antivimentin at 4°C. Primary antibodies, CK14 and vimentin, were diluted in PBS (1 : 200 and 1 : 100, resp.). Samples were then washed 3 times in PBS with 5 min for each time, incubated in FITC or Rhodamine-conjugated goat anti-mouse IgG (dilution 1 : 50) for 1 h at room temperature and washed again with PBS. For nuclei observation, samples were immersed in DAPI solution (3 *μ*g/mL in PBS) and rinsed with PBS. Immunofluorescence was observed using confocal laser scanning microscopy (CLSM, Olympus Fluoview-1000). Cell keratin or cytoplasm was displayed green or red fluorescence whilst nuclei were displayed blue.

### 2.8. *In Vivo* Biocompatibility of PEU Scaffolds

Female Wistar rats (3 months old, 250–300 g) were given an analgesic (ketoprofen 1.5 mg) and anesthetized with 5% chloral hydrate (intraperitoneal injection, 6 mg/kg) prior to implantation of sterile PEU and PEU-SF scaffolds which was cut into circular pat with diameter of 6.4 mm and thickness of ~2.0 mm. The rats were kept at Animal Test Center of Ningbo University. At the time point, that is, 20 d, 40 d, and 100 d, rats were anesthetized with 5% chloral hydrate and the samples were explanted with a small amount of surrounding tissue. For hematoxylin and eosin (HE) staining, samples were fixed in 4% paraformaldehyde at 4°C. Fixed samples were rinsed with water, dehydrated through a graded series of alcohol, made transparent with dimethylbenzene, wax-dipped, embedded in paraffin, sectioned at 4 *μ*m, dewaxed, hydrated, and stained with H&E dye. The stained samples were observed under light microscopy (Olympus CX40, Japan). Images were captured by digital camera (PL-B623CU, Pixelink, Canada).

All animals used in this study were treated under NIH Principles of Laboratory Animal Care of Ningbo University.

## 3. Results and Discussion

### 3.1. Characterization of PEU Scaffolds

**Figure 1:**
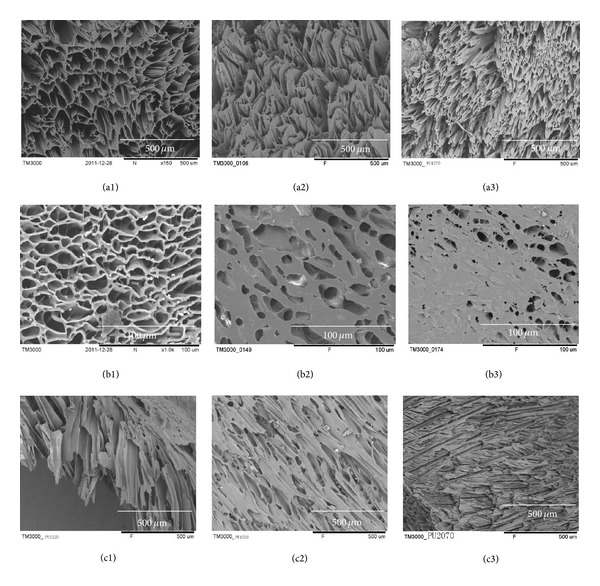
The morphology of PEU scaffold observed under SEM. The scaffold was prepared from PEU/DMSO solution with concentration of 10% (w/w) (1), 15% (2), and 18% (3), respectively, followed by quenching at −70°C. (a) Top face (contacting air during preparation), (b) bottom (contacting container during preparation), and (c) cross-section face.

Normal hypopharynx tissue is composed of four layers, that is, mucosa, fibrous layer, muscularis externa, and adventitia. The mucosa is the innermost tissue layer and comprises stratified squamous ECs which grow on a basement membrane (BM). The BM serves as the ECM to modulate ECs' function including adhesion, proliferation, differentiation, and migration. In order to remodel these histological features and support coculture of ECs and fibroblasts, an asymmetrical PEU scaffold with pores of 1–20 *μ*m in diameter on one side surface and ≥200 *μ*m on the opposite side and in the bulk was fabricated using phase separation method. The concentration of polymer solution usually plays an important role in pore formation. In this study, the effects of polymer concentration from 5% to 18% on the pore properties of the scaffold were explored.  Figure 1 showed the scaffold morphology with polymer concentration ranging from 10% to 18% observed under SEM. When the polymer concentration increased, pore size on scaffold top face (Figure 1(a)) and in the bulk (Figure 1(c)) decreased obviously. Because the crystal generation rate and the total number of DMSO crystals shall increase with the increase of the concentration and viscosity of the polymer solution, smaller pores were formed when the solution was quenched at −70°C and dried in a freeze dryer. Conversely, crystal formation rate reduced but crystal size increased when the polymer solution was diluted; therefore, larger pores were generated. However, the scaffold will collapse when polymer concentration was lower than 10% (w/w). On the other hand, the pores at the bottom face (contacting with the glass container during preparation) showed to be fine first at and then fused to produce sparse and uneven pores as polymer concentration increased from 10 to 18% (Figures 1(b1)–1(b3)). Based on these findings, scaffolds for subsequent experimentation were prepared in a 10% PEU/DMSO solution, quenched at −70°C, and dried in freeze dryer for 24 h. Using a liquid displacing method [20], the porosity of this PEU scaffold is calculated to be 76.2 ± 5.9%.

**Figure 2:**
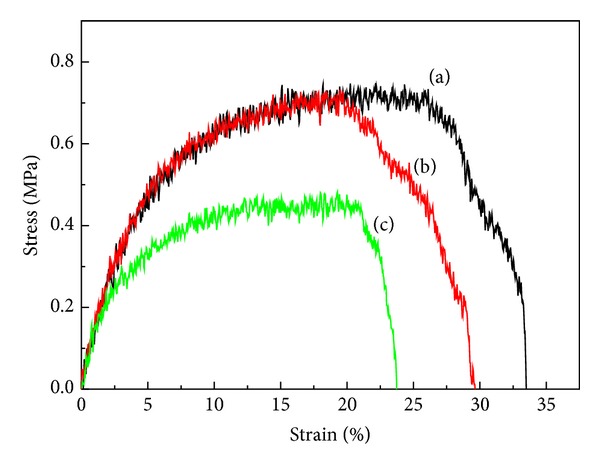
Tensile stress-strain curves for PEU scaffold fabricated from polymer solution concentration of 10% (a), 15% (b), and 18% (c).

The mechanical properties of these scaffold samples were tested.  Figure 2 is the tensile stress-strain curve of scaffolds generated from three different concentrations, that is, 10, 15, and 18%. The scaffold's mechanical properties were affected by the polymer solution concentration under the same quenching process. The PEU scaffolds made from 10% concentration exhibited the best strength and strain (0.7 ± 0.03 MPa and 23.2 ± 2.5%, Figure 2(a)) among all scaffolds, though the differences between them were slight. We had tested the mechanical property of submucosa tissue of porcine upper esophagus. The ultimate strength was 4.25 ± 1.40 MPa for untreated submucosa and 1.00 ± 0.45 MPa for submucosa decellularized with phospholipase and DNase [22], both of which were stronger than that of the present porous scaffolds. Thus, we chose the scaffolds made from 10% concentration as the biocompatibility-testing candidate.

**Figure 3:**
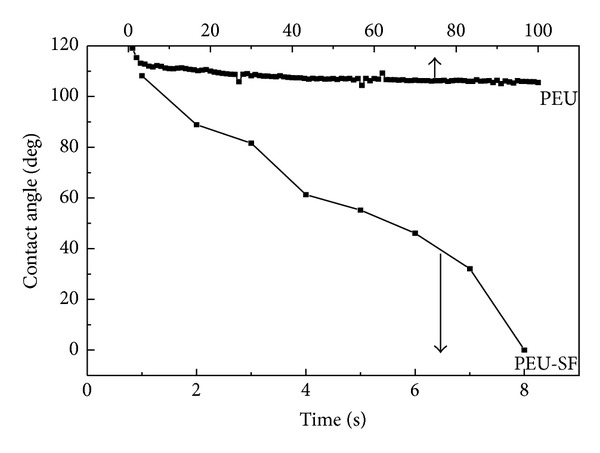
Dynamic contact angle of PEU and PEU-SF as a function of time. The contact angle was measured in ambient atmosphere at 25°C.

Considering the poor hydrophilicity of PEU material, we grafted protein SF onto the scaffold's surface using diamine aminolysis and GA crosslinking method aiming to improve its cell compatibility. The method of diamine aminolysis and GA crosslinking had been applied in our group for surface modification of ester-containing polymeric materials [23–25]. The material's surface was firstly aminolyzed with diamine to form a covalent bond, –CONH–, while the other unreacted amino group of the diamine was provided to react with one aldehyde group from GA molecule. Proteins-containing amino groups can be grafted via the reaction of the other aldehyde group from GA and amino group from protein. The density of amino group and the surface chemistry had been characterized and quantified to confirm the reaction occurrence and protein grafting. In this work, PEU is an ester-containing polymer. The ester group from PEU molecule on the scaffold surface reacts with one amino group from 1,6-hexanediamine. The other amino was kept pendant to react with one aldehyde group from GA. Finally, proteins like SF were grafted onto PEU surface via reaction between aldehyde from GA and amino group from SF. The aldehyde group of GA would not react with imine group from PEU molecules at 4°C and normal pressure. After modification, the surface's hydrophilicity was tested via dynamic contact angle measurements.  Figure 3 showed that the dynamic contact angle (CA) of PEU-SF decreased dramatically and reached minimal degree in 8 seconds. Conversely, the CA value of control PEU changed slowly from 120° to 106° in 100 seconds. Improvement in wettability verified the occurrence of SF grafting reaction, which was expected to enhance cell-material interactions [26].

### 3.2. Cytocompatibility of PEU Scaffolds

**Figure 4:**
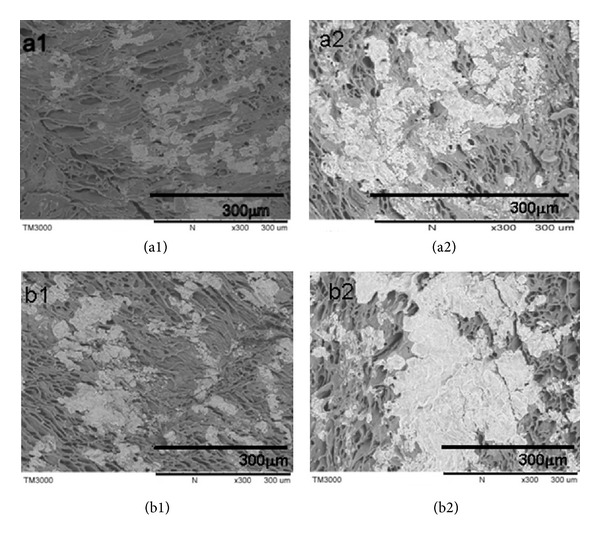
The morphology of epithelial cells which were cultured on the scaffold of control PEU (a) and PEU-SF (b) for 7 d (1) and 14 d (2), respectively. The scaffold was cut to fit in 96-well culture plate. Cells were cultured at 37°C in humidified air with 5% CO_2_ (the same conditions were followed for all cell culture). The seeding density is 5 × 10^5^/mL and 50 *μ*L cell suspension was used for each well. Samples were observed under SEM.

In order to optimize coculture of two cell types on/in the scaffolds, cytocompatibility to primary ECs and fibroblasts was evaluated separately at first. For ECs, morphological observation and immunofluorescence assay were carried out after cells were cultured on scaffold surfaces for 7 d and 14 d. Epithelial cells are usually slower to attach and grow than other cell types like SMC and fibroblasts, both of which are also present in hypopharynx tissue [27]. On the control scaffold, a little scattered ECs were attached to the microporous face at day 7. After culture for 14 d, more cells were evident due to cell proliferation; however, the general cell density remained low (Figures 4(a1) and 4(a2)). In comparison, the cell number on PEU-SF scaffold was greater after 7 d and 14 d of culture (Figures 4(b1) and 4(b2)).

**Figure 5:**
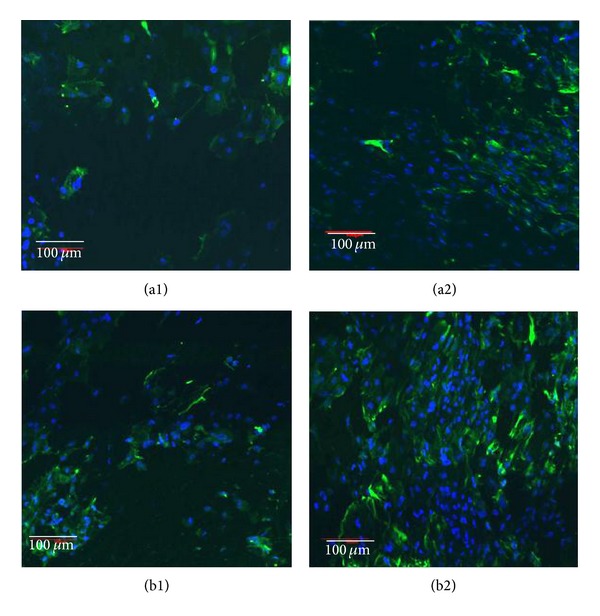
Immunofluorescence of epithelial cells cultured on PEU (a) and PEU-SF (b) for 7 d (1) and 14 d (2), respectively. Cell cytoplasm displayed green from CK-14 antibody and nucleus displayed blue from DAPI staining under CLSM observation. The seeding density is 5 × 10^5^/mL and 50 *μ*L cell suspension was used for each well. Scale bar 100 *μ*m.

**Figure 6:**
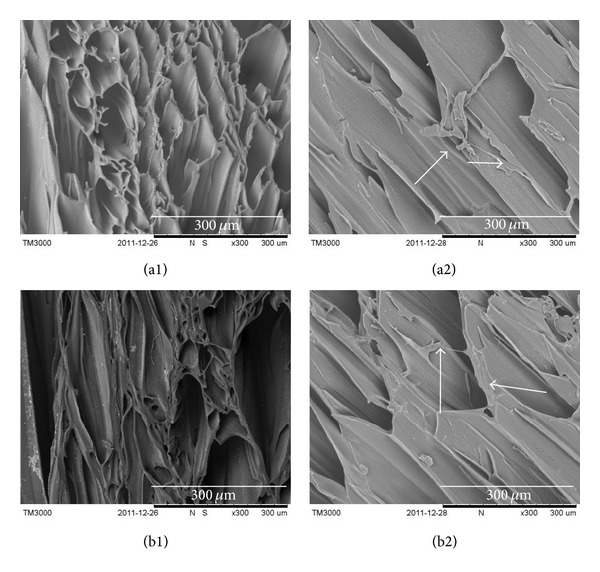
The morphology of fibroblasts which were cultured in PEU (a) and PEU-SF (b) for 14 d. (1) Top face; (2) cross-section of the scaffold. Samples were observed under SEM. The seeding density is 5 × 10^5^/mL.

**Figure 7:**
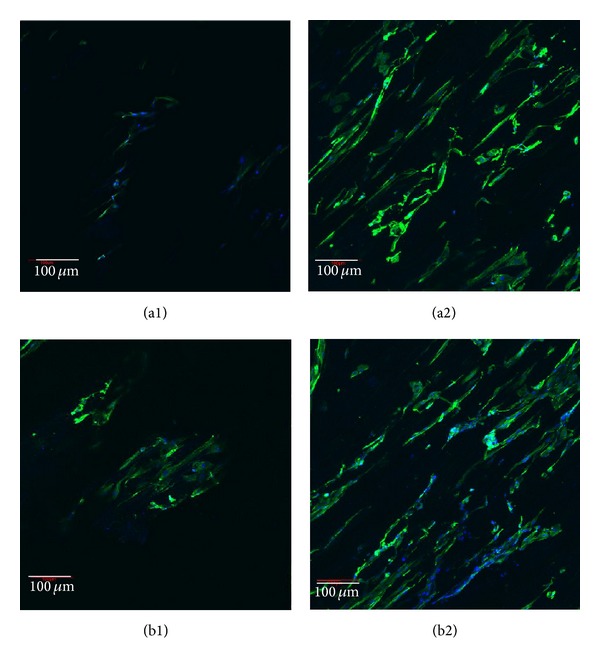
Immunofluorescence of fibroblasts seeded in the bulk (cross-section) of PEU (a) and PEU-SF (b) for 7 d (1) and 14 d (2), respectively. Cell cytoplasm displayed green from vimentin antibody and nucleus displayed blue from DAPI staining under CLSM observation. The seeding density is 5 × 10^5^/mL and 50 *μ*L cell suspension was used for each well. Scale bar 100 *μ*m.

Keratin is intermediate filament and serve is as the major cytoskeletal component of ECs [28]. Cytokeratin 14 (CK14) is a 50 kD polypeptide expressed by the basal cell of squamous epithelia, some glandular epithelia, myoepithelium and mesothelial cells, and so forth [29]. Using anti-CK14 as a primary antibody, cells cultured on PEU and PEU-SF scaffolds were immunostained to observe the morphology and confirm epithelial origin (green fluorescence). From the green fluorescence and nucleic number (blue) at day 7, there were more cells that lived on PEU-SF than on PEU, which hinted more cells survived on PEU-SF during the first 7 d culture (Figures 5(a1) and 5(b1)). After culture for 14 d, cells almost covered the whole PEU-SF scaffold surface (Figure 5(b2)). The general cell number on PEU-SF shall exceed the PEU scaffold (Figure 5(a2)). However, PEU material is sure to be nontoxic for EC because there were still many ECs that lived after 14 d culture. We therefore conclude that the grafted SF promotes the growth of hypopharyngeal epithelial cells. Moreover, PEU material is nontoxic and biocompatible, which is consistent with what some literature reported [30]. Hypopharynx fibroblasts were seeded in the bulk through the large pores of the scaffold. Under SEM observation, some fibroblasts scattered in the scaffold bulk in cross-section image (arrow) after cells were cultured for 14 d (Figures 6(a2) and 6(b2)) though it seemed very blur on top faces of both control and PEU-SF scaffolds (Figures 6(a1) and 6(b1)). In order to detect the cells embedded in the scaffold bulk, we stained the cells using antivimentin as the primary antibody and observed the cross-sectioned samples under confocal laser scanning microscope (CLSM). Vimentin is the most frequently found intermediate filament in fibroblasts. It is a reliable fibroblast marker. After the immunofluorescence staining, a few fibroblasts were distributed in the scaffold when cells were cultured for 7 d (Figures 7(a1) and 7(b1)). More cells appeared when cultured for 14 d (Figures 7(a2) and 7(b2)). Cells were proliferated and exhibited the fibroblast phenotype. These results indicated that fibroblasts could grow into the scaffold bulk using the present seeding and culturing methodologies.

### 3.3. Coculture of Primary ECs and Fibroblasts

**Figure 8:**
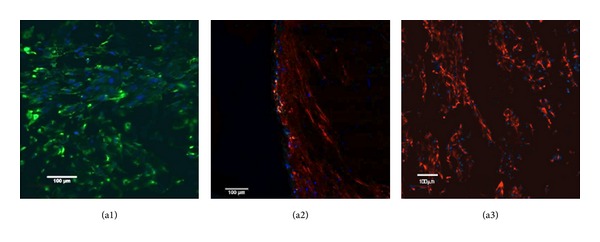
Immunofluorescence of cocultured epithelial cells (green) and fibroblasts (red). (a1) and (a3) both are surfaces (bottom and top), and (a2) is cross-section. Epithelial cells were seeded on scaffold bottom surface at the density of 5 × 10^5^/mL after fibroblasts were seeded through scaffold's top face to the bulk at the density of 5 × 10^5^/mL for 7days. 50 *μ*L cell suspension was used for each cell type. The coculture was conducted for another 14 d. Cell cytoplasm of epithelial cell and fibroblast was stained green and red from CK-14 and Vimentin antibody, respectively. The double staining (green first and then red) was performed for the cross-section (a2). The nucleus displayed blue from DAPI staining. The observation was conducted under CLSM. Scale bar 100 *μ*m.

In order to mimic the natural constitution of hypopharynx tissue, primary ECs and fibroblasts, both originating from the same hypopharynx tissue and the same passage, were cocultured into PEU-SF scaffolds. After fibroblasts were seeded in the scaffold for the first 7 d, ECs were subsequently seeded on the scaffold microporous surface and kept in the co-culture for another 14 d. The culture was evaluated using immunofluorescence analysis.  Figure 8 showed the morphologies of cells on the surface and in the bulk of PEU-SF scaffolds. The surface with micropores was lined with ECs (Figure 8(a1), green) and the scaffold bulk was filled with fibroblasts (Figure 8(a3), red).  Figure 8(a2) displayed the cross-section, in which ECs were stained green and fibroblasts were stained red. But the green fluorescence was overlaid by red fluorescence due to the red color domination. This architecture was similar to the constitution of natural hypopharyngeal tissue, where squamous ECs line the mucosa layer and neighbor the fibroblast-containing connective tissue.

### 3.4. *In Vivo* Biocompatibility of PEU Scaffolds

**Figure 9:**
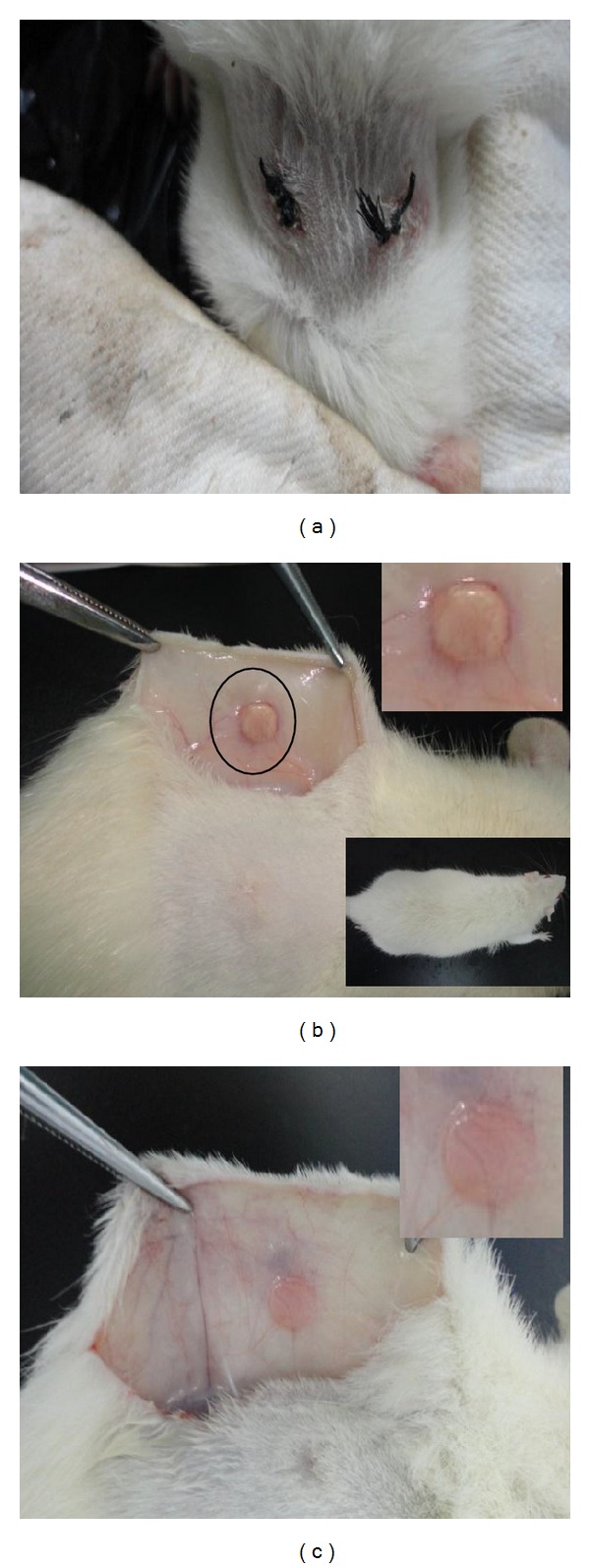
Overview images after rat was anatomized. PEU (b) and PEU-SF (c) were implanted in Wistar rat subcutaneously for 40 d. (a) is the appearance of live rat after scaffolds were implanted for 3 d. The lower inserted image showed the operated wound recovered and the fur regenerated completely after scaffolds were transplanted for 20 d. The upper inserted images in (b) and (c) showed the scaffold overview to display the vascularization.

Cell-free PEU-SF scaffolds were implanted subcutaneously into Wistar rats to detect their *in vivo* biocompatibility using control PEU as the comparison (Figure 9(a), two cuts on rat's back). The wound was observed to recover completely and the fur regenerated normally at day 20 (Figure 9(b) lower inserted), though inflammation occurred during the first three days after the operation (Figure 9(a)). After 40 d, the control PEU scaffold was completely encapsulated in a tissue bag and vascularization was apparent; blood vessels went through the tissue bag (Figures 9(b) and 9(c) upper inserted). However, the scaffold was observed to protuberate under the membrane, lacking the integration with neighboring tissue. In comparison, PEU-SF was more homogeneous with the surrounding rat skin, accompanied with some angiogenesis (Figure 9(c)). These results implied that PEU-SF was more biocompatible to animal tissue than the PEU scaffold.

**Figure 10:**
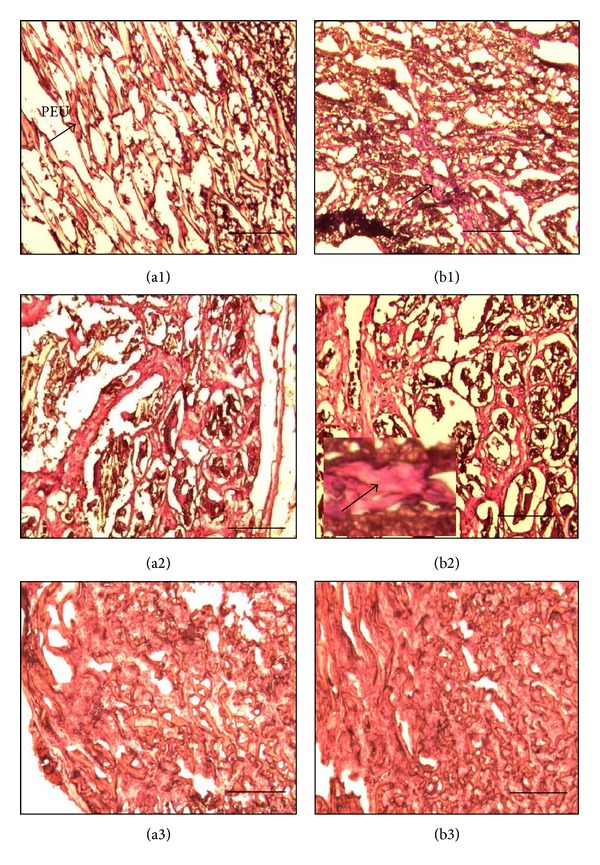
Hematoxylin and eosin (H&E) staining. Scaffolds PEU (a) and PEU-SF (b) were implanted subcutaneously into Wistar rat back for 20 d (1), 40 d (2), and 100 d (3), respectively. The inserted images were of higher magnification. The arrow pointed out the growing tissue in scaffold. Scale bar 200 *μ*m.

In order to investigate tissue infiltration into the scaffold, scaffolds were histologically analyzed by H&E staining. At day 20, fewer fresh tissues (red) were infiltrated into the scaffold pores of both PEU and PEU-SF scaffolds (Figures 10(a1) and 10(b1), arrow; yellow is PEU material). More fresh tissues infiltrated into scaffolds for both PEU and PEU-SF scaffold with the time passing by. Comparatively, more tissues grew in PEU-SF than in PEU at the same time point (Figures 10(b) versus 1(a)), which might be attributed to more hydrophilic and better cytocompatible of PEU-SF scaffold due to the SF grafting. At day 100, fresh tissue almost suffused the whole scaffold bulk while PEU material was not very visible (Figure 10(b3)). The scaffold was degradated while fresh tissue overgrew. The degradation of PEU material has been detected in our previous study, where the material was made as a solid membrane and SF grafting was performed on membrane surface [17]. After being subcutaneously implanted into rat back, the scaffold degradation occurred on SF-grafted membrane surface at day 20 but on ungrafted membrane at day 40; some crannies and small fragments emerged on the membrane surface. At day 150, one material layer was eroded and peeled off from the grafting membrane. Herein, SF grafting and porous structure promoted tissue infiltration and material biodegradation.

## 4. Conclusions

An asymmetrical porous 3D scaffold was designed and fabricated using phase separation method with DMSO as the porogen. The scaffold comprised pores of 1–20 *μ*m in diameter on one side and ≥200 *μ*m on the opposite side and in bulk, mimicking the mucosa constitute of hypopharyngeal tissue. After surface grafting with natural SF, scaffolds exhibited good hydrophilicity, which enhanced the interaction between cells and material. Primary porcine hypopharyngeal ECs were seeded on the scaffold's micro-porous surface and fibroblasts were seeded in scaffold bulk through the macropores. After coculture for 14 d, a confluent EC layer and 3D fibroblast patch were produced. *In vivo* investigation verified that PEU material possessed low toxicity and good biocompatibility. SF grafting greatly improved scaffold's hydrophilicity and thus enhanced cell/tissue infiltration. We therefore concluded that PEU porous scaffolds fabricated from the present protocol were able to support cogrowth of primary ECs and fibroblasts. SF grafting greatly promoted scaffold's *in vitro* cytocompatibility and *in vivo* biocompatibility. Evaluation of *in situ* hypopharynx regeneration is being designed with the cooperation of doctors from the affiliated hospital of our school, using porcine as the model animal.
